# Dementia incidence among a cohort of lebanese older adults: first incidence estimates from the Middle East and North Africa region

**DOI:** 10.3389/frdem.2024.1494719

**Published:** 2025-01-07

**Authors:** Monique Chaaya, Sarah Assaad, Hala Darwish, Marc Haber, Rosemary Khoury, Dahlia Saab, Khalil El Asmar, Ramzi Hajjar

**Affiliations:** ^1^Department of Epidemiology and Population Health, American University of Beirut, Beirut, Lebanon; ^2^Research Department of Epidemiology and Public Health, University College London, London, United Kingdom; ^3^Department of Neurology, University of Michigan School of Nursing, Ann Arbor, MI, United States; ^4^Division of Neurosurgery, American University of Beirut, Beirut, Lebanon; ^5^Department of Internal Medicine, American University of Beirut Medical Center, Beirut, Lebanon

**Keywords:** dementia incidence, Alzheimer's disease, MENA region, incidence rates, gender differences, regional differences, lebanese older adults

## Abstract

**Objective:**

To provide the first estimates of dementia incidence rates among elderly in Lebanon.

**Methods:**

The cohort established in 2013 consisted of 470 elderly from Beirut and Mount Lebanon, who consented to be followed-up. In 2017, we reached 341 participants/informants, achieving a response rate of 72.6%. The validated Arabic version of the 10/66 Dementia Research Group diagnostic tool was administered through face-to-face interviews. Direct age standardization was applied to the data using the Western European population distribution. Age-, sex- and location-specific incidence rates were estimated.

**Results:**

After 3.5 years of follow up, 19 new cases of dementia were identified among 229 surviving participants. The crude incidence rate was 16.8 per 1,000 p-y, and the age standardized rate was 20.5 per 1,000 p-y. The incidence rate increased with age, going from 6.5 for those aged 65–74 years to 54.0 for those aged 85–89 years. Incidence rate was higher among females than males (20.7 vs.12.0), and higher in Mount Lebanon, as compared to the capital city Beirut (19.5 vs.14.9).

**Conclusion:**

Dementia incidence rate was close to European and North American countries' estimates. The use of validated tools increased the internal validity of our results. A large cohort study is warranted to confirm these results.

## 1 Introduction

Dementia represents a significant public health challenge, exerting both emotional and financial tolls on society at large, and on individuals in particular. According to the most recent data from the Global Burden of Disease study, dementia contributes to almost 5% of total disability-adjusted life years (DALYS) for individuals aged 70 years and above globally (IHME, 2021). Worldwide, 55 million live with dementia, and this number is expected to increase to 139 million by 2050 (WHO, 2021). Notably, the prevalence is higher among women as compared to men (8.1% and 5.4%, respectively) (WHO, 2021). High-income countries are experiencing a decreasing trend in age-specific dementia incidence (Matthews et al., 2016; Qiu et al., 2013; Schrijvers et al., 2012); however, the global burden continues to escalate due to aging populations (Satizabal et al., 2016).

The increase in the aging population in the Middle East and North Africa (MENA) region is consistent with the global trend (Yaghmour et al., 2019). However, in 10 countries (48%) within the MENA region, there is notable lack of information regarding dementia burden or risk, indicating knowledge and awareness deficit about dementia in this region (Bhalla et al., 2018; El-Metwally et al., 2019). The only study reporting on dementia incidence in the MENA region is a systematic review. The evidence indicates a scarcity of available data on incidence rates, with a significant proportion of countries in the region needing more information on dementia (Bhalla et al., 2018).

Lebanon presents a unique case study for dementia research. The country's diverse population genetic makeup, its distinct socio-economic and cultural context, and the high proportion of older adults (10.8%, the highest in the region) provide crucial insights into the dementia risk (Saxena, 2008). A 2013 cross-sectional study in Lebanon found a 9.0% age-standardized prevalence of dementia in individuals aged 65 years and above (Phung et al., 2017). This prevalence exceeds worldwide estimates, suggesting a potentially crippling burden of dementia in Lebanon (Qassem et al., 2023).

This study aims to estimate the overall dementia incidence rate in Lebanon, as well as age- and gender-specific rates, to address the gap in knowledge of dementia incidence in the MENA region.

## 2 Methods

### 2.1 Study design

The Cohort of Lebanese Elderly: a Dementia Study, or COLDS for short, is a prospective population-based cohort study among elderly aged 65 years and above. Data were collected at two timepoints: baseline data was collected in 2013 with follow-up assessments conducted in 2017. Interviews were administered in the participants' homes.

### 2.2 Study population and sample

**Figure 1:**
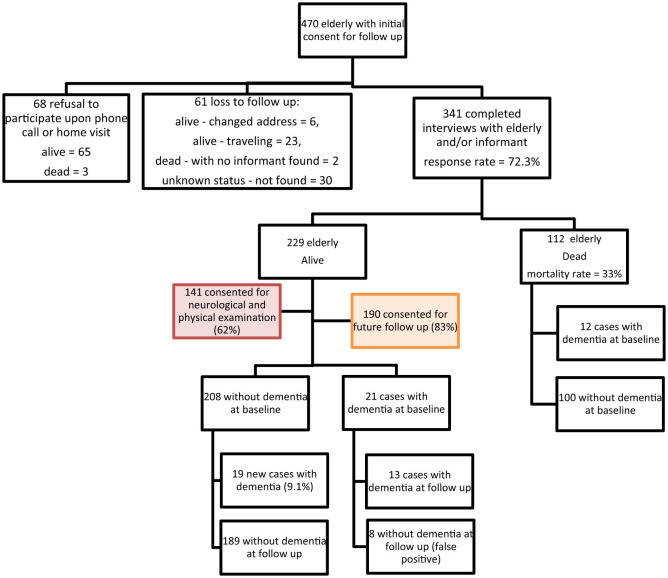
Cohort study flow chart.

The study was conducted in two of the eight governorates of Lebanon: (i) Beirut, the capital, characterized by its dense urban setting and crowded neighborhoods and (ii) Mount Lebanon (Shouf and Aley districts; two out of six districts in Mount Lebanon), a peri-urban district. Study participants were recruited using a multi-stage random sampling approach based on the Lebanese population distribution data from the latest census. Initially, we divided Lebanon into major administrative regions, selecting Beirut and Mount Lebanon (Shouf and Aley districts) to represent urban and peri-urban areas, respectively. Using a sampling frame from another survey, Beirut governorate was divided into 594 clusters, each containing 50 residential buildings, with complete detailed household listing for 60 randomly selected clusters. Since there was no existing sampling frame in Chouf and Aley districts, villages and towns were randomly chosen and weighed based on their respective sizes. All households within the randomly selected clusters were approached and within each household, eligible individuals (aged 65 and above) were identified, and one participant per household was randomly selected for inclusion. This method ensured coverage of diverse household types. At baseline 502 elderly were interviewed and assessed for dementia. Of those, 470 gave their initial consent for a follow-up visit, and 341 were included in the follow-up interviews (Figure 1). Of these, 19 were diagnosed with dementia. During the 2017 follow-up, eight cases initially classified as dementia were identified as false positives upon re-evaluation. This misclassification may have been influenced by testing limitations, including hearing impairments in two participants and the 10/66 Dementia Research Group diagnostic tool while having high specificity (92%), has a false positive rate of up to 8%, especially among those with no formal education. The result is 328 elderly at risk of dementia.

### 2.3 Diagnosis of dementia

Arabic validated one-stage 10/66 Dementia Research Group (DRG) diagnostic assessment tools were used (The 10/66 Dementia Research Group, 2015). These tools include (i) the community screening instrument for Dementia (CSI-D), (ii) the modified Consortium to Establish a Registry of Alzheimer's Disease (CERAD) animal naming tests and modified 10-word list recall, (iii) the CSI-D informant interview, (iv) the physical assessment, (v) the Geriatric Mental State (GMS) examination test, and (vi) brief neurological examination (NEUROEX). The algorithm, initially developed by Prince et al. (2003), yields a binary outcome for each older adult to assess dementia status (Phung et al., 2014).

### 2.4 Statistical methods

We calculated the age-specific incidence rate by 5-year age intervals. We divided the number of cases by the number of person-years (p-y) which was calculated by summing up every participant's contribution of follow-up duration per age interval. The follow-up period ended at the date of the follow-up interview, which was considered as the age of dementia onset for positive cases. Incidence per 1,000 p-y was estimated as the number of new cases of dementia divided by the at-risk p-y multiplied by 1,000. The incidence rates presented in this study reflect a follow-up period of 3.5 years, spanning from 2013 to 2017. This time frame defines the observation window and scope of measured dementia incidence in the cohort. We calculated incidence rates per 1,000 person-years to account for person-time at risk, ensuring a clear representation of dementia risk over time in our cohort. Confidence intervals were calculated assuming a Poisson distribution for the number of cases within each reporting category. The standardized incidence rate of dementia was reported using direct age standardization based on the Western European population distribution. We applied standardization using the Western European population distribution to enable comparability with global dementia incidence studies.

### 2.5 Ethical considerations

The Institutional Review Board (IRB) at the American University of Beirut approved the study (protocol # FHS.MC.34). A decision-making capacity test was conducted before every interview to ensure the participants' comprehension of the study. Written consent was secured from both participant and his/her informant and in the presence of a witness in case the participant is unable to give written consent. All participants were offered brochures that highlight the common symptoms of dementia individuals and essential information for the caregiver on how to care for the persons living with dementia. In addition, a list of health care centers that provide affordable primary care services was also provided.

## 3 Results

Table 1 displays a comparison of the socio-demographic profile between the 341 participants included in the follow-up cohort and the 129 participants who lost to follow-up. The majority of the follow-up sample was recruited from Beirut (60%), were females (57%), were under 75 years of age (60%), were married (72%) and have completed some school education (72%). As compared to the follow-up sample, a significantly higher proportion of those who were lost to follow-up were residents of Beirut district, younger than 75 years old, married, and had some school education.

**Table 1:** Socio-demographic profile of the follow-up and the loss to follow-up samples.

	**Cohort with completed interviews**, ***N*** = **341**		**Loss to follow-up (including refusal)**, ***N*** = **129**		***P*-value^a^**
	***n***	**%**	***n***	**%**	
**District**					
Beirut	205	60	94	73	0.010
Shouf	68	20	23	18	
Aley	68	20	12	9	0.006
**Gender**					
Female	194	57	69	53	
Male	147	43	60	47	
**Age group**					
67 to < 75 years	206	60	105	81	< 0.001
75 to < 85 years	108	32	19	15	< 0.001
85+ years	27	8	5	4	
**Marital status**					
Never married	15	5	2	2	
Married	192	58	94	74	0.002
Widowed/divorced/separated	124	37	31	24	0.008
**Educational level**					
No formal education	78	23	13	10	0.002
School education	245	72	103	80	
University or higher	18	5	13	10	
**Evaluation of income**					
Insufficient	89	27	34	27	
Sufficient	239	73	93	73	
**Occupation**					
Not working^*^	237	71	82	65	
Working	95	29	45	35	

^*^Retired/housewife or husband.

^a^z-test of proportions, only significant values at 0.05 were reported.

The characteristics of the study sample are summarized in Figure 1. Initially, 470 participants consented to a follow-up visit, and among them, 341 (73%) were included in the cohort study, out of which 112 (33%) died during the follow-up period. Dementia diagnosis was performed for 229 subjects, out of which 190 consented for a third follow-up visit. After a mean follow-up period of 42 months ± 2.7 (3.5 years ± 0.22) and 1130 person-years follow-up, 19 new dementia cases were identified.

**Figure 2:**
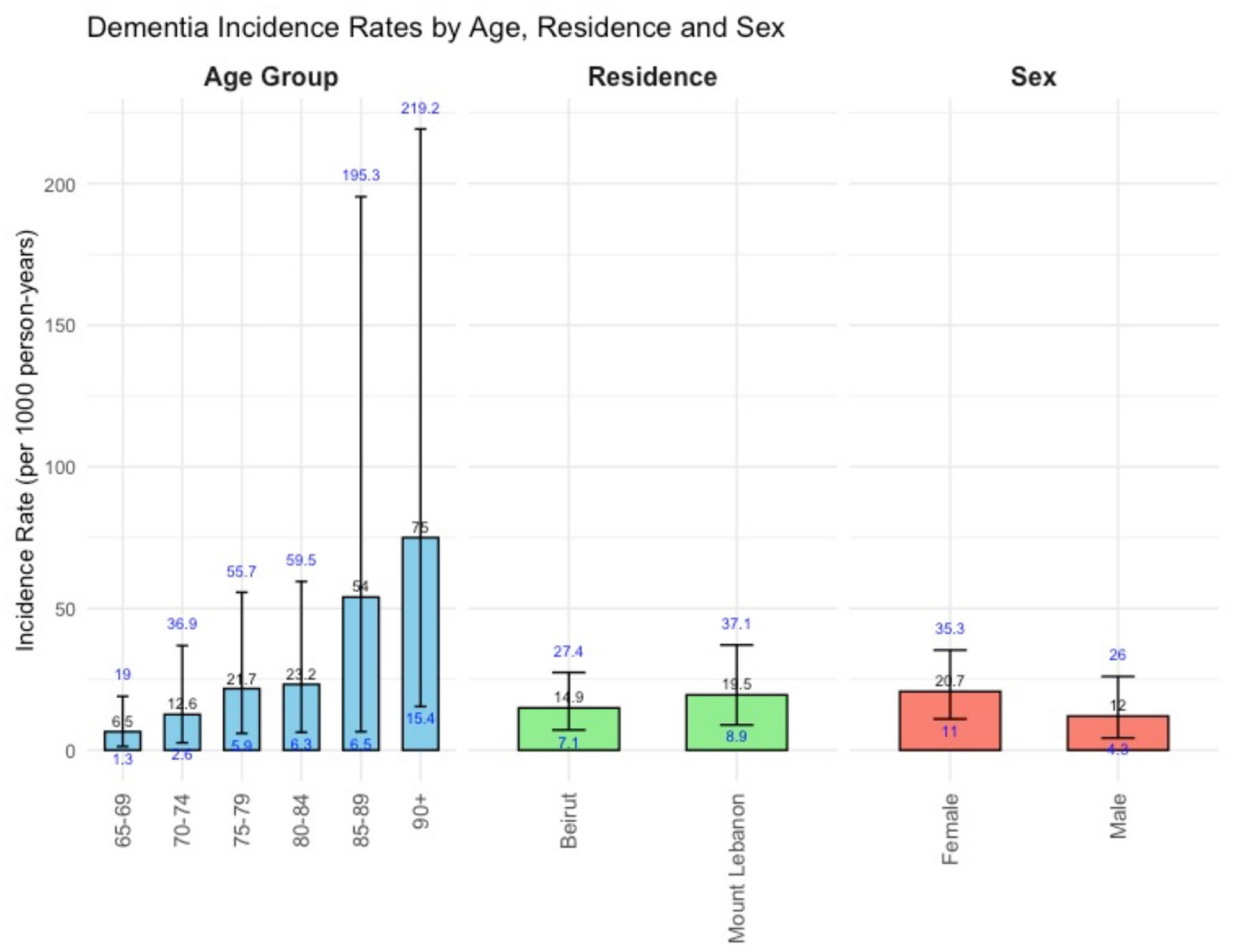
Bar plot illustrating dementia incidence rates (per 1,000 person-years) with 95% CI by age, gender, and location.

**Figure 3:**
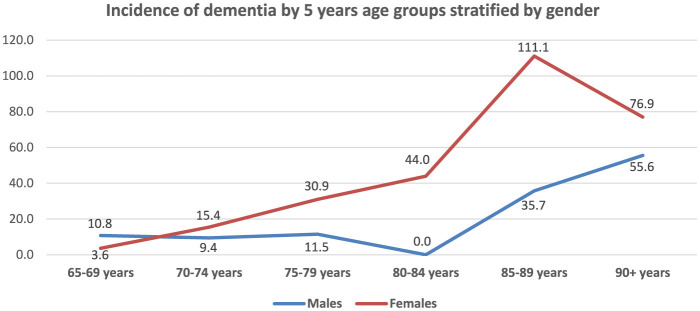
Age and gender-specific incidence of all dementia.

Figure 2 presents age, gender, and location-specific incidence rates of dementia. The overall incidence rate was 16.8 per 1,000 p-y [95% CI: (10.1; 26.2)], and the standardized incidence rate was 20.5 per 1,000 [95% CI: (13.5; 27.5)]. The incidence rate of dementia showed a sharp increase with age ranging from 6.5 per 1,000 p-y [95% CI: (1.3; 19.0)] at the ages of 65–69 years to 75.0 per 1,000 p-y [95% CI: (15.4; 219.2)] at age 90 and older. The age-specific incidence rates (Figure 3), increased steeply for women as compared to men after 80 years of age, but dropped back to closer rates (55.6 per 1,000 for men and 76.9 per 1,000 for women) at the age of 90.

The incidence rate in Mount Lebanon was 19.5 per 1,000 p-y [95% CI: (8.9; 37.1)], higher than Beirut's 14.9 per 1,000 p-y [95% CI: (7.1; 27.4)], with overlapping confidence intervals.

## 4 Discussion

The estimated dementia incidence in our study is comparable to that reported in European and North American populations. However, further research with larger samples and extended follow-up periods is essential for confirming this pattern in the Lebanese context. The standardized incidence rate is 20.5 per 1,000 person-years, higher than the global age- and gender-standardized incidence of 17.30 per 1,000 person-years for those aged 60 and above reported in the World Alzheimer Report 2015 (Prince et al., 2015). It is also higher than rates observed in Low and Middle-Income Countries (LMICs) and Latin America, which were 14.06 and 15.11 per 1000 person-years, respectively (Prince et al., 2015). The high incidence rate of dementia in Lebanon might be attributed to several factors. One possibility is the use of a higher age cut-off when estimating the incidence rate which was 5 years higher compared to other low-to-middle income countries. Additionally, the high prevalence of uncontrolled hypertension and other cardiovascular risk factors in the older age group may contribute (Gauthier Serge et al., 2022). Specifically, 35% of elderly in Lebanon experience uncontrolled hypertension (Fakhri et al., 2020). Moreover, the widespread lifestyle factors, such as smoking and dietary habits (Alzheimer's Disease International, 2023; Jeong et al., 2023), may also play a role; 70.9% of Lebanese adults are smokers (Nakkash et al., 2022), and a notable proportion follow unfavorable dietary patterns (Hoteit et al., 2024). Furthermore, the high consanguinity rate, which was reported as 35.5% in Lebanon in 2009, may also influence disease occurrence (Barbour and Salameh, 2009). Additionally, differences in methodologies and case identification may also contribute to differences in incidence rate from other published estimates.

It is difficult to compare our study's incidence rate to the few estimates available from the Arab region (Bhalla et al., 2018). The source population for the dementia cases used in the numerators of the previously cited incidence rate estimates was unclear, making it difficult to interpret accurately. Moreover, these estimates did not consider individuals' time contributions to the risk of dementia, which is an important factor. Our study followed dementia-free participants for 3 years, contributing updated incidence rate data from the Arab region. However, divergent methodologies used across studies limit conclusions about trends.

According to various studies conducted globally, the incidence rate trend of dementia varies with age, and our study aligns with those trends. For instance, the World Alzheimer Report of 2015 showed that the incidence rate of dementia doubled every 6.3 years, ranging from 3.9 to 104 per 1,000 person-years for ages 60–64 to 90+ years (Prince et al., 2015). Similarly, our study found that the age-specific incidence rate of dementia in LMICs and Latin America was comparable to other studies using the 10/66 DRG tools, which reported 18.2 to 30.4 dementia cases per 1,000 person-years (Prince et al., 2015, 2012).

Multiple studies have indicated that women are at a higher risk of developing dementia compared to men. For instance, a study of 16,926 Swedish twins, half of whom were women, revealed that women had higher rates of dementia incidence for all types of dementia (Beam et al., 2018). Likewise, a 27-year study of dementia incidence trends across Europe and the United States discovered that while men experienced a 24% decrease in incidence, women only experienced an 8% decrease (Wolters et al., 2020). This smaller decrease in incidence among women exacerbates the already substantial burden of dementia on them. In addition, women are more prone to psychological problems, like depression, than men (Eid et al., 2019), and female brains are influenced by sex hormones which create higher risk factors for developing Alzheimer's disease (Zhu et al., 2021). In addition, women tend to have a longer life expectancy than men (Tower, 2017), making them an aging population with increased risk of developing dementia.

Although the number of events in our study is relatively low, the consistency of the observed trend in LMICs on age, and gender-specific incidence estimates reinforce the reported findings.

A scant literature exists concerning regional variations in dementia incidence, with more emphasis historically placed on prevalence. However, some studies have examined rural-urban differences. A nationwide study in Taiwan reported higher dementia prevalence in rural (8.69%) vs. urban areas (4.46%) (Liu et al., 2022). Similarly, a study in the United States found higher Alzheimer's disease incidence in rural compared to metropolitan counties (Rahman et al., 2021). Additionally, a systematic review reported worse dementia care quality and outcomes in rural areas (Arsenault-lapierre et al., 2023). Those findings align with those observed in our study which also reports higher dementia incidence rates in less urbanized areas. This pattern suggests that geographical factors might influence the epidemiology of dementia. Our analysis revealed significant educational disparities between Beirut and Mount Lebanon, where 36.03% of participants from Mount Lebanon lacked formal education, compared to only 14.15% in Beirut. Furthermore, 81.46% of Beirut participants had school-level education, while this figure was markedly lower (57.35%) in Mount Lebanon. Such disparities in foundational education levels may contribute to the observed differences in dementia incidence, as lower education is associated with reduced cognitive reserve, a factor known to impact dementia onset (Darwish et al., 2018; Wilson et al., 2019). The cognitive reserve theory posits that education enhances the brain's resilience to cognitive decline, thereby delaying dementia onset. Therefore, the lower overall educational attainment in Mount Lebanon may partly explain the higher dementia incidence in this region. Future studies should explore educational influences on dementia in more depth, particularly in settings with similar urban-rural educational gaps.

### 4.1 Limitations

The study's limitations stem from a restricted participant and dementia case pool, focusing solely on two out of eight governorates in Lebanon, which makes the findings less applicable beyond the sample and reduces the generalizability of the results. The original cohort was established to assess the feasibility of following up with older adults in Lebanon and document any challenges. This led to the creation of a larger cohort, which is part of an ongoing, more extensive cohort study design called the “Lebanon Study on Aging and Health” (Lebanon Study on Aging Health, 2024). However, factors such as loss of follow-up and participant mortality might have led to an underestimation of dementia incidence rate. The study had a response rate of 72.3%, with 129 participants lost to follow-up due to changes in address, travel, or refusal to participate when contacted for the second interview. This could introduce selection bias, which might have affected the estimate of dementia incidence rate. To assess potential biases from attrition, we compared the sociodemographic characteristics of retained participants and those lost to follow-up. We documented the differences to ensure that the reported dementia incidence rate was not affected by non-responding participants' data. Selection bias is likely to have partially explained the high incidence rate of dementia in the cohort. Compared to the retained participants, we found that a significantly higher proportion of those lost to follow-up were from urban areas (Beirut), aged 67–75 years, and formally educated. Although we applied standardization using the Western European population distribution, we recognize that a future standardization based on the Lebanese population structure could offer additional insight specific to the local context.

### 4.2 Strengths

According to the World Alzheimer Report, there is a lack of data on dementia incidence from regions including Australasia, Asia Pacific, South Asia, sub-Saharan Africa, and the Middle East. In their meta-analysis, only 5% of person-years came from these regions (Prince et al., 2015). Our study is significant because it provides the first dementia incidence rate estimates in the Arab region using the validated tools from the 10/66 Dementia Research Group, which have been tested in diverse Lebanese populations. Despite the small sample size, the incidence rate found is similar to that of other low and middle-income countries, indicating accurate observed data.

## 5 Conclusion

There is a scarcity of longitudinal studies on dementia in developing countries, with none specifically focusing on the Middle East and North Africa (MENA) region. This study addresses this gap by presenting the inaugural estimates of dementia incidence rates in Lebanon using a cohort design, contributing crucial data for the MENA region. The study reveals that dementia incidence rates increase with age, which aligns with the global trends. Furthermore, women have slightly higher incidence rates than men, and the incidence rates vary by region. These baseline incidence rates and demographic patterns will serve as a valuable foundation for researchers to conduct future studies on dementia etiology and risk factors in Lebanon and the region. The next phase of the study will involve a larger cohort that covers more areas of Lebanon to gain a fuller understanding of dementia epidemiology in this population and allow for a more detailed analysis of gender and regional variations in the dementia incidence in Lebanon.

## Data Availability

The raw data supporting the conclusions of this article will be made available by the authors, without undue reservation.
